# Oxytocin Administration in High-Intensity Focused Ultrasound Treatment of Myomata

**DOI:** 10.1155/2018/7518026

**Published:** 2018-07-02

**Authors:** Tomasz Lozinski, Justyna Filipowska, Piotr Krol, Anna Kubaty, Piotr Wegrzyn

**Affiliations:** ^1^Specialist Hospital Pro-Familia, Rzeszow, Poland; ^2^Chair of Electroradiology, Institute of Nursing and Health Sciences, Faculty of Medicine, University of Rzeszow, Poland; ^3^Department of Obstetrics, Gynecology and Oncology, The Gabriel Narutowicz Hospital, Krakow, Poland; ^4^Department of Obstetrics and Perinatology, Faculty of Health Sciences, Medical University of Warsaw, Zwirki i Wigury Str. 63a, 02-091 Warsaw, Poland

## Abstract

**Objectives:**

The aim of the study was to evaluate the clinical efficacy of magnetic resonance-guided High-Intensity Focused Ultrasound (HIFU) in patients with symptomatic uterine fibroids (myomata) after application of oxytocin.

**Methods:**

156 women with symptomatic uterine fibroids were treated using MR-guided HIFU procedure. 51 patients had additional IV administration of 40 IU of oxytocin in 5% Glucose or 0,9% NaCl solution during therapy. Before and after the procedure we performed MR and measured initial perfused volume, final perfused volume, nonperfused volume (NPV), and treated volume ratio (TVR). The follow-up was up to 15 months to assess efficacy of treatment and relief of symptoms.

**Results:**

Nonperfused volume was statistically significantly larger in oxytocin group than in control group (p=0.0019). The remaining parameters did not show significant difference between both groups.

**Conclusion:**

Oxytocin administration seems to improve efficiency of HIFU therapy although further research is required to assess its value. This study' clinical registration number is DRKS00014794.

## 1. Introduction

The uterine fibroid (also known as fibromyoma or myoma) is the most common benign disease in women [1–3]. Symptoms caused by fibroids, e.g., heavy bleeding, anemia, pain, increased size of the abdomen, and specific fertility and pregnancy complications, are major causes of morbidity in women in reproductive age. They occur in at least 40% of all women and account for significant healthcare and social costs due to surgical treatment (mainly hysterectomies) and subsequent absence at work [4]. The presence of fibroids can lead to decrease of fertility due to impairment of endometrial receptivity and abnormal implantation of the blastocyst. It has been suggested that fibroids may also disrupt normal myometrial peristaltic movements impeding sperm arrival at the fallopian tubes and embryo transport into the uterus [5, 6].

The fibroids can be classified by as subserosal, intramural, or submucosal depending on their location in the uterus. Pedunculated fibroids arise from either the serosal surface or from the mucosal surface. Histologically they are benign, hormone-sensitive smooth-muscle tumors containing fibrous connective tissue. They are demarcated from the surrounding uterine tissue by a pseudocapsule. Fibroids can undergo hyaline degeneration or hemorrhagic infarction. After menopause they regress and may calcify [3].

The purpose of the fibroid treatment is relief of symptoms such as pain and bleeding. There are many therapeutic methods available. Historically first- and last-line therapy was a surgical procedure: myomectomy or hysterectomy. Myomectomy can be associated with penetration to the uterine cavity that induces adhesion, subsequently leading to a decrease in fertility. Hysterectomy causes irreversible sterilization. The presence of uterine fibroids is the most common indication for hysterectomy in the United States [4]. Operative treatment is still gold standard for symptomatic fibroids. Each case should be considered very carefully based on individual variables. Optimal treatment depends on age, symptoms, parity, and further reproductive plans. Many patients are afraid of surgery and its consequences and want to avoid or even decline this method of treatment. Unfortunately, alternative treatment options are very limited.

Medical therapy is essentially a treatment option for the control of symptoms including reduction in fibroid volume and in menstrual blood loss. Oral contraceptive pills administration decreases symptoms such as pain and bleeding but no pharmacological agent is curative of fibroids. Unfortunately OTC does not work in a certain population of patients, so this option is limited even as a symptom relief treatment. GnRH analogues have been most commonly used drugs. Recently new agents have shown promising effects in symptom improvement and fibroid regression, like aromatase inhibitors, mifepristone, selective estrogen receptor modulators, and selective progesterone receptor modulators. They do not cause hypoestrogenic symptoms associated with GnRH analogues [5].

Less invasive procedures such as laser therapy, artery embolization, and cryoablation are safer than surgical treatment but unfortunately they also have certain limitations. Laser ablation of fibroids can be performed though laparoscopic or endoscopic route or using percutaneous approach under the magnetic resonance (MR) guidance. Interstitial laser photocoagulation, with low-power laser guided by thermal monitoring, is used to destroy the fibroid tissue. A 1064 nm wavelength laser is applied to ensure sufficiently deep penetration into the fibroid tissue. Laparoscopic or hysteroscopic laser ablation has been shown to decrease fibroid volume by 50%–70% [7]. Cryotherapy can be applied through laparoscopic or hysteroscopic approach. Low temperature is obtained by rapid freezing using high-pressure gas. Side effects of this procedure are infection end fever [2]. Radiofrequency ablation is used through laparoscopy or hysteroscopy. The drawback of this technique is the lack of possibility of monitoring the local temperature in the threated tissue [2].

MR-guided High-Intensity Focused Ultrasound (HIFU) procedure is a noninvasive option of fibroids therapy. In 1942, Lynn et al. proposed the use of Focused Ultrasound (FUS) to achieve thermal effect in tissue. Device to treat neurologic disorders such as Parkinson's diseases was created in 1950 by Fry Brothers [8]. A research about utility of MR-HIFU for another disease has taken many years. At the beginning in 21st century uterine fibroids started to be treated by ultrasound beam [7]. InSightec Exablet system 2000 received FDA approval in 2004 for fibroids treatments. Up to now, many patients with symptomatic fibroids were treated. This method of conservative treatment of symptomatic uterine fibroids has been reported to be effective and safe [9]. Another system that has been approved in Europe is Sonalleve MR-HIFU (Philips Healthcare, Andover, MA, USA).

MR-controlled ultrasound beam causes heating of the tissue and temperature-related protein denaturation in the tumor tissue. Clinical symptoms of fibroids lessen relatively quickly but decrease of fibroid volume takes much more time. [9]

## 2. Objectives

The aim of the study was to assess the influence of supplementary IV oxytocin administration on HIFU treatment in women with symptomatic uterine fibroids. The application of MR-HIFU therapy is limited to certain group of patients, depending on the type of fibroids, their localization, and positive qualification based on MR criteria. [9, 10]

In our clinical practice, in open surgery and laparoscopic procedures, we observed beneficial effects of in- and post-surgery oxytocin administration, e.g., less bleeding and shorter time of Douglas pouch catheter drainage. Other researches reported similar to observations. [11, 12]

## 3. Material and Methods

156 women with symptomatic uterine fibroids were enrolled to this study between September 2016 and June 2017. In 2016 we did not use oxytocin. We started recruiting consecutive patients to the oxytocin group from the beginning of 2017. There were no differences including and excluding factors in both groups of patients. Each patient was counseled about potential risks and side effects of oxytocin and signed informed consent form.

Types of fibroids according to FIGO classification are shown in Table 1 [13].

Willingness to preserve fertility, history of pregnancy loss, and infertility associated with fibroids were main inclusion criteria. We also included asymptomatic patients with fibroids that were not able to conceive spontaneously for more than 12 months and/or with history of miscarriages. Other inclusion criteria were as follows: age 20-43 years (mean 36.3), symptomatic fibroid or fibroids (≤2), and positive MR qualification (Funaki type I or II, suitable beam window). Mean BMI was 23.2.

Exclusion criteria were as follows: standard contraindications for MR imaging, age >43 years, and unwillingness to preserve fertility.

All patients were treated using MR-guided HIFU procedure with Sonalleve MR-HIFU (Philips Healthcare, Andover, MA, USA). HIFU was performed in Specialist Hospital Pro-Familia, Rzeszow.

The ultrasound beam has to have an unobstructed access to the fibroid. If the bowel loops were in front of the uterus it was necessary to change position of the bowel by filling the bladder with saline solution and/or the rectum with ultrasound gel. Otherwise it would not be possible to perform the procedure [14]. The amount of subcutaneous fatty tissue is also a very important factor. The distance between the tumor and skin should not be more than 13 cm [14, 15].

In the oxytocin group there were 51 patients and 105 in the control group. In the first group 40 IU of oxytocin diluted in 500mL of 5% glucose or 0,9% NaCl was administered during the whole procedure by IV catheter at the rate of 5mL/min. Every 30 minutes blood pressure and heart rate measurements were taken and no differences were found as compared to the control group. Patients reported no complaints. No side effects were reported after oxytocin administration.

Before the procedure MR was performed to measure mean fibroid volume and initial perfused volume (IPV). Right after the procedure treated volume ratio (TVR) and final perfused volume (FPV) were assessed on MR, and subsequently nonperfused volume (NPV) was calculated (NPV=FPV-IPV). At follow-up MR scan 6 months after the procedure mean fibroid volume change was measured.

After checking for the normality of the data distribution, the* t*-test was performed to determine if the results are significantly different from each other in both groups. The difference was considered statistically significant if p-value was <0.05. Statistical analyses were done using Statistica Software (Version Stat Soft. Inc., Tulsa, OK, USA).

Ethical Approval was received from Local Ethics Committee (Polish Medical Chamber, Rzeszow Department). Clinical registration number is DRKS00014794, URL: https://www.drks.de/drks_web/navigate.do?navigationId=trial.HTML&TRIAL_ID=DRKS00014794.

## 4. Results

Average time of the whole treatment was 220 min, time of sonication 111 min, 60 s, and patient's preprocedure preparation such as obtaining optimal position, filing the bladder, and/or the rectum another 80 minutes. Average volume of fibroids was 90 ml.

T2-weighted hypointense fibroids showed a frequency of 93.6%; isointense and hyperintense fibroids had frequencies of 5.60% and 1.1%, respectively. There was a negative correlation between NPV and age (r = -0.083, p = 0.307) and treatment time (r = -0.253, p = 0.001). Median TVR was 96.0 % in small and 76.5 % in large fibroids.

**Figure 1:**
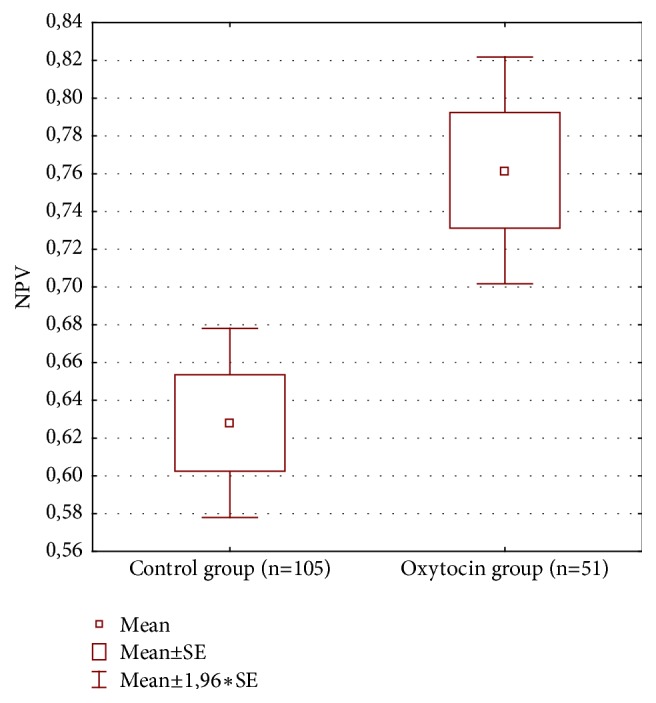
Nonperfused volume (NPV) in both groups.

**Figure 2:**
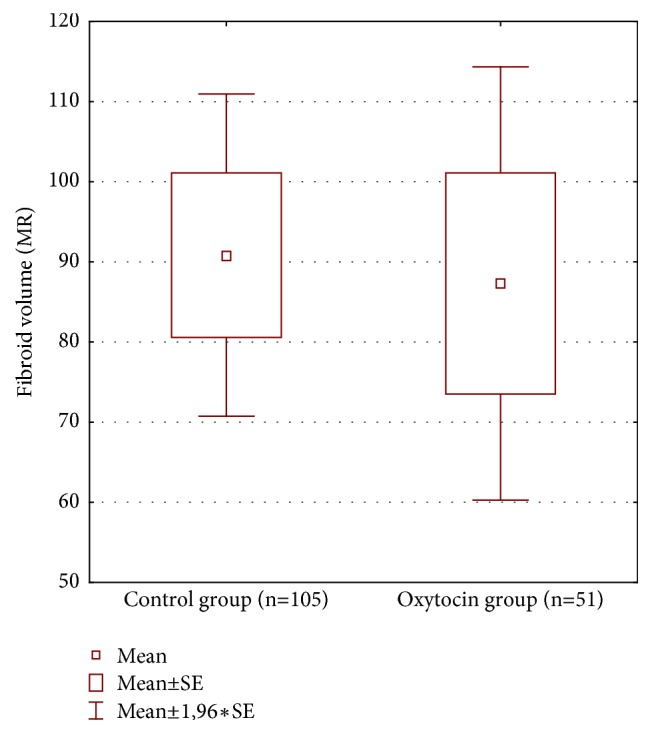
Fibroid volume in both groups.

**Figure 3:**
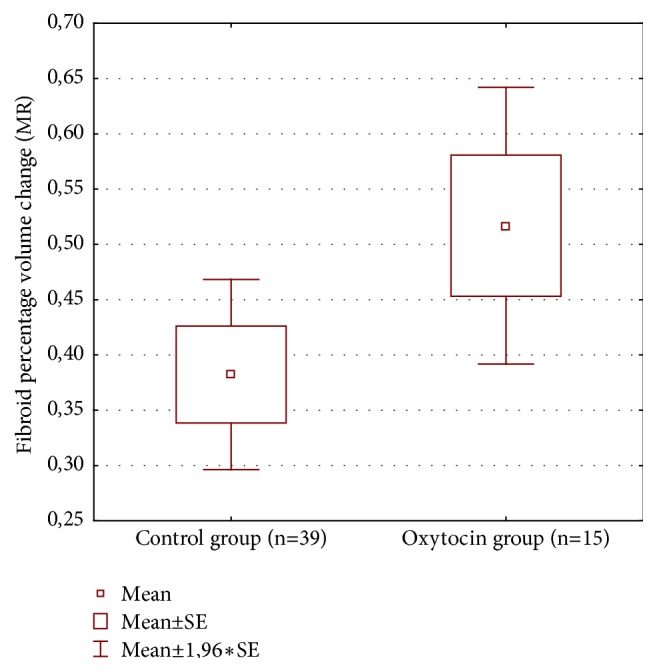
Fibroid volume change in both groups.

We found a significant difference in NPV that was larger in patients with oxytocin administration during HIFU therapy (Table 2 and Figure 1). No significant difference was found in mean fibroid volume (Table 2 and Figure 2). Mean fibroid volume change (Table 2 and Figure 3) was larger in the oxytocin group but the difference was not statistically significant possibly due to small number of patients with this parameter assessed at follow-up (15 versus 39).

## 5. Discussion

Oxytocin is nonapeptide hormone released by the hypothalamus, causing contractions and involution of the uterus and closure of uterine blood vessels after delivery. Estrogens act synergistically with oxytocin. Stimulation of receptors situated on the cervix and nipples induces release of oxytocin. Application of oxytocin is widespread in the world in induction of labor. It was believed that oxytocin does not have an effect on nonpregnant uterus, because there are no detectable oxytocin receptors in the myometrium. However high-dose oxytocin infusion during myomectomy decreases blood loss during the procedure [11, 12]. This is consistent with our observation of decreased blood loss after oxytocin administration during laparoscopic and open surgery myomectomy alike. This data will be published soon.

We found that NPV (nonperfused volume) was significantly larger in patients with oxytocin administration during HIFU therapy. Also the mean fibroid volume change (decrease of volume as measured in MR) was noticeable but the difference has not reached the level of statistical significance, possibly due to a small number of patients with this parameter assessed (15 in oxytocin group versus 39 in control). This requires further research with follow-up in larger group of patients.

Our data suggests that oxytocin has some effect for nonpregnant uterus. Contraction of uterine muscle and blood vessels decreases blood flow and that may lead to better results in HIFU therapy. Funaki found that efficiency of HIFU depends on vascularity and flow in tumor [10].

The practical consequence of oxytocin administration during HIFU was an increase of efficiency of the HIFU treatment. NPV is a parameter quantifying the degree of necrosis after treatment. Therefore measurement of NPV allows assessing effectiveness of the treatment. Our results showed that NPV was significantly better after oxytocin administration.

It could be hypothesized that oxytocin causes contraction of the fibroid leading to externalization of extracellular fluid and, as a result, more effective heating of the tumor tissue by the ultrasound beam. An increase in the blood flow in the fibroid tissue decreases the efficiency of HIFU treatment. This phenomenon is known as a “cooling effect”. We suspect that oxytocin administration decreases this effect due to vasoconstriction; hence heating of the tumor tissue is increased [16]. Similar observation about the oxytocin effect during MRgFus was first reported by Zhang et al. [17].

The average duration of the MF-HIFU is longer than the surgical treatment, but from our experience no patient considered it as a drawback. Zhang observed shorter duration of the procedure and better NPV in patients stimulated by oxytocin during treatment of adenomyosis [17]. Jeong reported the same observations in patients with fibroids [18].

## 6. Conclusions

Intravenous administration of oxytocin during MR-HIFU therapy may potentially improve efficiency of fibroid treatment. However it still requires further research.

## Figures and Tables

**Table 1:** Types of fibroids according to FIGO classification [13].

**FIGO type**	**1**	**2**	**3**	**4**	**5**	**6**

**Oxytocin group (N=51) **	6	1	21	11	7	5

**Control group (N=105)**	9	5	36	22	16	17

**Table 2:** MR parameters in both groups.

	**Oxytocin group (N)**	**Control group (N)**	**Oxytocin group**	**Control group**	**t**	**df**	**p**
**Mean fibroid ** **volume (mL)**	51	105	87.31	90.84	0.201	154	0.8411

**NPV (%)**	51	105	76.2	62.8	-3.153	154	**0.0019**

**Mean fibroid ** **volume change (%)**	15	39	51.7	38.2	-1.659	52	0.103
